# Insomnia and internet addiction: a longitudinal examination using random intercept cross-lagged panel modeling

**DOI:** 10.1186/s40359-025-03795-6

**Published:** 2025-12-11

**Authors:** Hanlin Ren, Junhua Dang, Jie Liu, Hongyu Zou

**Affiliations:** 1 School of Humanities and Social Sciences, Xi’an Jiaotong University, Xi’an, China; 2 The Third People’s Hospital of Zhongshan, Zhongshan, China; 3 Department of Surgical Sciences, Uppsala University, Uppsala, Sweden; 4 School of Psychology, South China Normal University, Guangzhou, China; 5 School of Medicine, Indiana University, Indianapolis, USA

**Keywords:** Insomnia, Internet addiction, Random intercept cross-lagged panel model

## Abstract

The increasing prominence of insomnia and internet addiction among university students has raised significant concerns, as these conditions substantially impair physical and mental well-being as well as academic performance. Understanding the dynamic interplay between these phenomena is critical for developing effective interventions. The longitudinal study investigated the bidirectional relationship between insomnia and internet addiction among college students through data collected from 313 participants assessed at three time points, spaced six months apart. The study applied random intercept cross-lagged panel models (RI-CLPM) to examine both between-person and within-person effects. At the between-person level, our findings revealed no significant correlation between insomnia and internet addiction. However, at the within-person level, a reciprocal predictive relationship was found: insomnia at one time point significantly predicted increased internet addiction at the next time point, and vice versa. These results highlighted the dynamic and mutually reinforcing interaction between insomnia and internet addiction over time, offering valuable insights into the processes underlying their co-occurrence. By using the RI-CLPM methodology, this study enhanced understanding of how fluctuations in insomnia and internet addiction occur at the individual level, moving beyond the understanding of them as trait-like phenomena. These results emphasized the importance of addressing both issues concurrently in interventions for college students.

## Introduction

Sleep is essential for humans, playing a vital role in regulating numerous physiological functions, maintaining good mental health, enhancing learning ability, and promoting overall health [56]. However, insomnia has emerged as a major public health concern [4]. According to the DSM-5, insomnia disorder is characterized by difficulty initiating sleep, difficulty maintaining sleep, and/or early-morning awakening, which causes clinically significant distress or impairment. This condition contributes to disrupted sleep rhythms, weakened immunity, and increased risks for various physical and psychological disorders [1, 7].

The prevalence of insomnia is alarmingly high across different populations. In China, a nationwide study revealed that approximately 59% of the general population experience insomnia, and 64% report poor sleep quality, suggesting that between 800 million and 900 million people suffer from varying degrees of sleep problems [48]. The college years, a crucial developmental stage, are often marked by academic pressures, social demands, and lifestyle changes that make students particularly vulnerable to sleep disturbances, including insomnia [51]. Empirical studies across diverse regions consistently report high prevalence rates of insomnia among university students. Yao et al. [52] found that nearly 30% of Chinese students exhibited mild-to-severe insomnia symptoms, while Mbous et al. [40] reported a similar rate of 26.4% among students in the Midwestern United States. Specific risk factors may exacerbate this problem; for example, Ghrouz et al. [21] showed that Indian students with low physical activity levels had short-term insomnia rates as high as 51.5%. These findings are consistent with a systematic review of South Asian universities reporting a pooled prevalence of 52.1% [14].

Among college students, insomnia can lead to a range of adverse outcomes, such as inattention in class, reduced learning efficiency, mood swings, heightened anxiety and depression, strained social relationships, and increased physical health problems [32, 58]. These issues not only threaten the quality of life for students but may also compromise their future careers and social development [22]. Clarifying the longitudinal relationship between insomnia and related behavioral factors is critical for identifying targets for intervention, enhancing academic performance, and safeguarding their physical and mental health.

Internet addiction is a significant factor contributing to insomnia among college students. Defined as excessive, compulsive, or poorly controlled internet use [54], internet addiction undermines intrinsic motivation, academic performance [15], and mental health, with sleep being particularly affected [32]. Research shows that college students with internet addiction are 4.39 times more likely to report poor sleep quality [47]. The Sleep Displacement Theory posits that problematic internet use, particularly at night, reduces the time available for sleep and disrupts sleep patterns [17]. Internet-addicted students are more likely to delay their bedtime, leading to poorer sleep quality [53]. Furthermore, engaging in stimulating online activities such as gaming or social media interactions makes it harder to wind down before sleep [29]. Additionally, prolonged exposure to blue light emitted by electronic devices suppresses melatonin secretion and disrupts biological rhythms, further delaying sleep and impairing its quality [12, 57].

Insomnia, in turn, may exacerbate internet addiction. While college students commonly turn to the internet to explore new social relationships and manage academic and social pressures [30, 41], poor sleep quality may intensify this reliance. Specifically, insomnia-related anxiety and depressive symptoms may drive students to seek temporary emotional relief through internet use [27]. Activities like gaming or social media provide an escape from real-world stressors and sleep difficulties [49]. However, this reliance can evolve into internet addiction, characterized by an inability to control online behavior despite negative consequences [31]. Therefore, although initially a coping mechanism, insomnia-driven internet use can spiral into addiction, which in turn disrupts sleep further, creating a vicious cycle where insomnia and internet addiction reinforce each other.

However, previous research on insomnia and internet addiction among college students has primarily relied on cross-sectional designs. Although these studies consistently report a strong correlation between the two constructs (for a meta-analysis, see Li et al., [35], they cannot establish the temporal ordering of effects or disentangle causal influence. Some researchers have attempted to address this issue by employing longitudinal designs using the cross-lagged panel model (CLPM; e.g., Yao et al., [52]. While informative, the CLPM has well-documented limitations: it conflates stable between-person differences (e.g., students who are generally more prone to insomnia also tend to report higher internet addiction) with dynamic within-person processes, thereby risking spurious conclusions about causality [38]. Moreover, the CLPM is susceptible to inflated error rates when stable variance is present, potentially exaggerating associations.

To address these limitations, scholars have advocated for the use of random intercept cross-lagged panel models (RI-CLPM; Berry & Willoughby [8, 38],. The RI-CLPM introduces random intercepts that capture all time-invariant, trait-like between-person variance and allows these intercepts to covary, thereby isolating true within-person fluctuations over time. This modeling approach provides a cleaner and more precise test of temporal reciprocity, enabling researchers to ask whether changes in insomnia from an individual’s own baseline predict subsequent changes in internet addiction, and vice versa.

Building on these advances, the aim of the present study was to apply the RI-CLPM to rigorously examine the bidirectional relationship between insomnia and internet addiction at the within-person level across three waves of data collection. By separating between-person from within-person variance, this approach allows us to characterize the dynamic interplay of these conditions more accurately and provides an empirical foundation for future mechanism-focused and intervention-oriented research.

## Materials and methods

### Participants and procedure

This study employed a repeated cross-sectional design, consisting of three waves of data collection conducted over one year (November 2022 to November 2023) to assess the mental health status of college students at a university in South China. At T1, 2,797 students participated (2,709 valid); at T2, 2,050 participated (1,745 valid); and at T3, 3,134 participated (2,694 valid). Across waves, 689 participants were matched between T1 and T2, and 636 were matched between T1 and T3 based on student IDs. For the longitudinal analyses, participants matched across all three waves formed a final cohort of 313 students (43 males; Mage = 19.56, SD = 0.78). Consistent with our previous analyses using any two adjacent waves, attrition analyses showed that dropout was random and non-systematic (see Peng & Zou [43, 61]).

At each wave, participants were recruited through cluster random sampling and completed electronic questionnaires via computer or mobile phone (wjx.cn). To ensure data quality, participants were required to be 18–24 years old and cognitively able to complete the survey. Cases were excluded if their reported age or grade level was inconsistent, if they failed a lie-detection item (e.g., “Please select C: I am an honest person”), or if their response time was shorter than eight minutes. Upon survey completion, participants received a personalized mental health feedback report, and those with more severe concerns were offered free counseling services. Participation was voluntary, written informed consent was obtained, and confidentiality was strictly maintained. No monetary compensation was provided.

This study adhered to the principles outlined in the Declaration of Helsinki and was approved by the Ethics Committee of the School of Psychology at South China Normal University (Ethics Approval Number: SCNU-PSY-2022-217).

### Measures

#### Insomnia

The Insomnia Severity Index (ISI) is a widely used instrument for assessing the severity of insomnia symptoms. It has been revised and translated into Chinese [5] and validated in various studies [59, 60]. The ISI has been frequently used to evaluate the sleep quality of Chinese university students [36]. The questionnaire consists of seven items, including, for example: “How much do you feel the sleep problem interferes with your functioning (e.g., fatigue, mood, concentration, memory, work)?” Responses are rated on a Likert scale, where 0 indicates “very satisfied” or “not at all worried/noticeable/interfering,” and 4 indicates “very dissatisfied” or “very much worried/noticeable/interfering.” The ISI yields scores ranging from 0 to 28, with higher scores indicating poorer sleep quality. In this study, the ISI demonstrated excellent internal consistency across the three measurement waves, with Cronbach’s alpha values of 0.89, 0.90, and 0.89, respectively.

#### Internet addiction

Young’s [54] Internet Addiction Diagnosis Questionnaire (IAD) was among the first tools developed to assess internet addiction. The questionnaire was later revised and translated into Chinese [16] and validated in various studies [53]. For this study, we used the Chinese version of the IAD to measure the degree of impairment in academic, social, and occupational domains caused by internet misuse or overuse. The questionnaire includes eight items. One example item is: “Do you feel preoccupied with the Internet (e.g., thinking about previous online activity or anticipating your next session)?” To increase score variability, subsequent versions of the scale replaced the original dichotomous yes/no response format with various Likert scale formats (e.g.,Andreassen et al., [3, 53]. The version used in this study employed a 6-point Likert scale, ranging from 1 = “extremely disagree” to 6 = “extremely agree.” The IAD yields scores ranging from 8 to 48, with higher scores indicating poorer internet addiction behaviors. The IAD demonstrated excellent internal consistency in this study, with Cronbach’s alpha values of 0.92, 0.94, and 0.94 across the three measurement waves.

### Statistical analysis

Descriptive analyses were conducted in SPSS 29.0, and longitudinal measurement invariance testing as well as RI-CLPM analyses were performed in Mplus 8.3. To minimize measurement error associated with multi-item scales, the parceling approach was employed [37]. Longitudinal measurement invariance of the study variables was examined using confirmatory factor analysis (CFA) through a four-step hierarchical procedure: the configural invariance model, which tested whether the factor structure was consistent across time points; the metric invariance model, which constrained corresponding factor loadings to equality; the scalar invariance model, which additionally constrained item intercepts; and the strict invariance model, which further constrained error variances [39].

Progression to the next level was undertaken only if the preceding level was confirmed, and model comparisons indicated no significant deterioration in fit, defined as ∆CFI < 0.010 and ∆RMSEA < 0.015 [13]. Data were then analyzed using a RI-CLPM to disaggregate between-person and within-person effects [24]. Gender and age were included as covariates due to established associations with the study variables; specifically, females generally report higher insomnia levels [7], males show greater vulnerability to internet addiction [28], and emerging adulthood is a high-risk period for both conditions [52]. Five nested RI-CLPM models were compared: an unconstrained baseline model; a model with all cross-lagged paths fixed to be time-invariant; a model with all autoregressive paths fixed to be time-invariant; a model with correlations between insomnia and internet addiction at T2 and T3 constrained to be time-invariant; and a final model with all cross-lagged paths, autoregressive paths, and T2–T3 correlations simultaneously constrained.

Models were estimated using maximum likelihood estimation with robust standard errors (MLR) to account for potential non-normal distributions of the data. Model fit was evaluated using χ², the comparative fit index (CFI), the standardized root mean square residual (SRMR), and the root mean square error of approximation (RMSEA), with acceptable fit defined as CFI >0.90 and RMSEA and SRMR < 0.08 [25]. Invariance constraints were retained when ∆CFI < 0.010 and ∆RMSEA < 0.015 relative to the less restricted model [13].

## Results

### Descriptive statistics

Table 1 presents the descriptive statistics for all variables and the correlations within and across measurement occasions. Pearson correlation analyses revealed significant positive correlations between insomnia at T1, T2, and T3 (*r*s = 0.47–0.65,* ps* < 0.001) and between internet addiction at T1, T2, and T3 (*r*s = 0.52–0.64, *p*s < 0.001). Insomnia and internet addiction were also significantly positively correlated at the same time points (*r*s = 0.32–0.41, *p*s < 0.001), indicating a high degree of stability in their relationship. Furthermore, significant positive correlations were observed between insomnia and internet addiction across time points (e.g., T1 → T2, T2 → T3; *r*s = 0.24–0.34, *p*s < 0.001), supporting the lagged relationships assumed in the cross-lagged design.

**Table 1:** Descriptive statistics and correlation matrix for variables over three time points

	M	SD	1	2	3	4	5	6
1 ISI T1	7.10	5.31						
2 ISI T2	6.45	5.12	0.65***					
3 ISI T3	5.51	4.68	0.47***	0.58***				
4 IAD T1	15.85	8.52	0.41***	0.31***	0.18**			
5 IAD T2	15.53	8.73	0.24***	0.32***	0.29***	0.59***		
6 IAD T3	13.78	8.04	0.21***	0.34***	0.39***	0.52***	0.64***	

** *p* <.01; *** *p* <.001

The Intraclass Correlation Coefficients (ICCs) were calculated for all study variables. The ICC for insomnia was 0.57 (95% CI [0.51–0.63]), indicating that 57% of the total variance was attributable to between-person differences, while 43% was due to within-person variation. Similarly, the ICC for internet addiction was 0.58 (95% CI [0.52–0.64]), suggesting that 58% of the total variance stemmed from between-person differences, with 42% attributable to within-person fluctuations.

### RI‑CLPM between insomnia and internet addiction

Following the invariance testing protocol detailed in the Methods, we observed no significant differences between the configural, metric, scalar, and strict invariance models for any variable, confirming strict invariance and supporting meaningful comparisons across the three measurement waves (see Table 2).

**Table 2:** Model fit and comparison for measurement invariance

Variable	Models	χ^2^	df	CFI	RMSEA	SRMR	Comparisons	ΔCFI	ΔRMSEA
ISI	M1: Configural invariance	135.01	72	0.970	0.053	0.047			
	M2: Metric invariance	144.40	80	0.970	0.051	0.050	M2-M1	0.000	− 0.002
	M3: Scalar invariance	173.58	88	0.960	0.056	0.050	M3-M2	− 0.010	0.005
	M4: Strict invariance	194.25	98	0.955	0.056	0.054	M4-M3	− 0.005	0.000
IAD	M1: Configural invariance	500.16	225	0.922	0.063	0.045			
	M2: Metric invariance	521.87	239	0.919	0.061	0.049	M2-M1	− 0.003	− 0.002
	M3: Scalar invariance	551.85	253	0.915	0.061	0.051	M3-M2	− 0.004	0.000
	M4: Strict invariance	587.99	269	0.909	0.062	0.060	M4-M3	− 0.006	0.001

As reported in Table 3, the most parsimonious model (Model 5) did not make the model fit significantly worse than an unconstrained model (Model 1) (i.e., ∆CFI < 0.010 and ∆RMSEA < 0.015). Thus, we fixed the cross-lagged paths, stability paths, and T2-T3 correlated changes to be time-invariant.

**Table 3:** Model fits and comparisons for random intercept Cross-Lagged panel models

Model fits					Model comparisons					
Models	*χ* ^*2*^	*df*	CFI	RMSEA [90% CI]		Δ*χ*^*2*^	*df*	*p*	ΔCFI	ΔRMSEA
M1	4.97	9	1.000	0.000 [0.000–0.037]						
M2	8.84	11	1.000	0.000 [0.000–0.050]	M2-M1	3.87	2	0.15	0.000	0.000
M3	8.99	11	1.000	0.000 [0.000–0.050]	M3-M1	4.02	2	0.13	0.000	0.000
M4	4.98	10	1.000	0.000 [0.000–0.028]	M4-M1	0.01	1	0.92	0.000	0.000
M5	14.66	14	0.999	0.012 [0.000–0.057]	M5-M1	9.67	5	0.09	− 0.001	0.012

Model 1 = unconstrained baseline model; Model 2 = model with all cross-lagged paths fixed to be time-invariant; Model 3 = model with all autoregressive paths fixed to be time-invariant; Model 4 = model with correlations between insomnia and internet at T2 and T3 fixed to be time-invariant; Model 5 = model with all cross-lagged paths, all autoregressive paths, and correlations between insomnia and internet at T2 and T3 fixed to be time-invariant

**Fig. 1:**
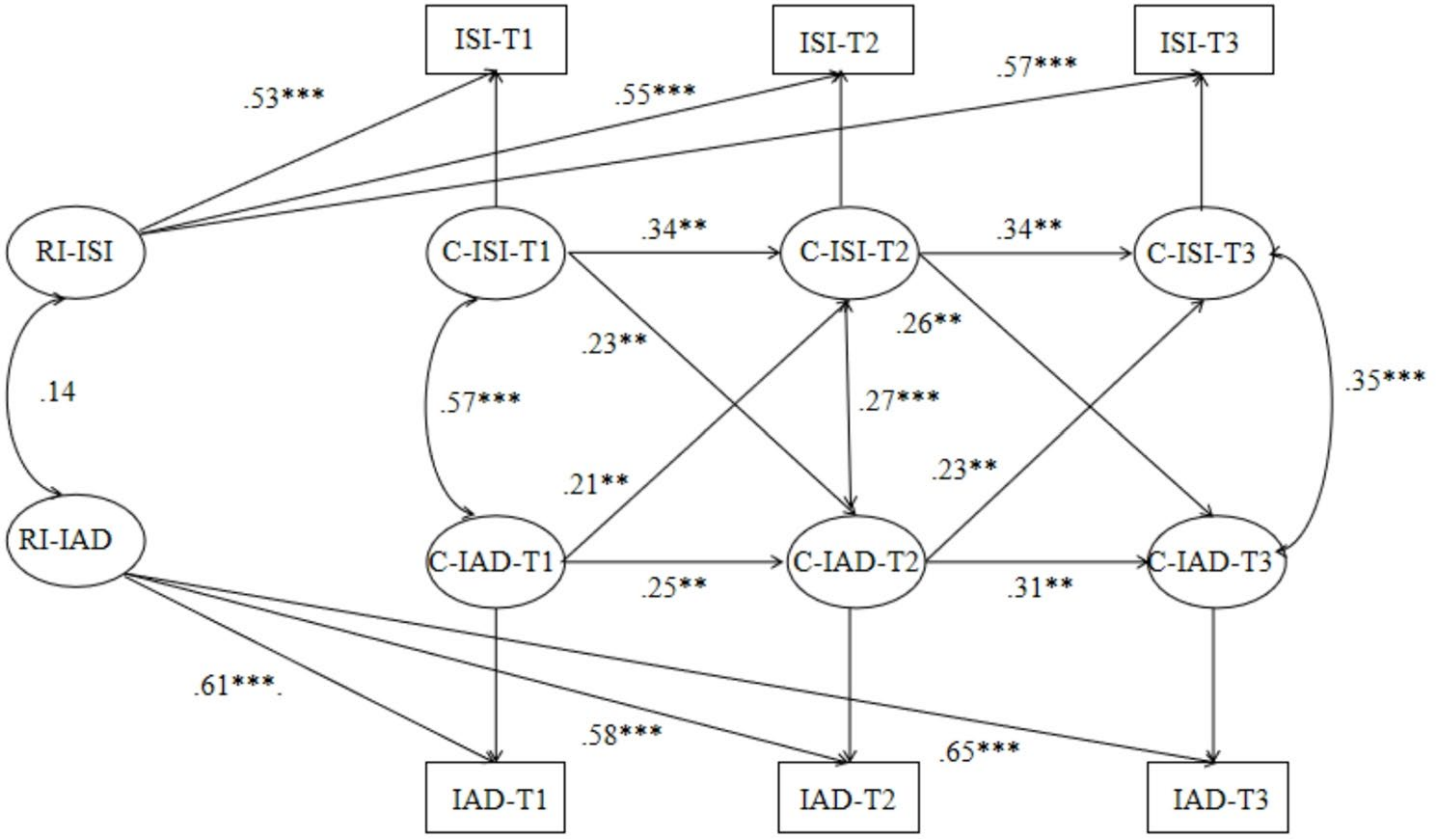
RI-CLPM for insomnia and internet addiction across three measurement occasions. *Notes*: The values in the figures are standardized path coefficients. RI_X stands for random intercept, ISI = Insomnia Severity Index, IAD = Internet Addiction Diagnosis Questionnaire. For the sake of clarity, paths from the control variable (i.e., gender and age) are not displayed. ** *p* < .01，***p < .001

As reported in Fig. 1, at the between-person level, the correlation between insomnia and internet addiction was not significant (*β* = 0.14, *p* =.631). At the within-person level, both insomnia and internet addiction showed significant positive autoregressive effects. Specifically, insomnia at T1 significantly predicted insomnia at T2 (*β* = 0.34, *p* =.001), and insomnia at T2 significantly predicted insomnia at T3 (*β* = 0.34, *p* =.001). Similarly, internet addiction at T1 significantly predicted internet addiction at T2 (*β* = 0.25, *p* =.001), and internet addiction at T2 significantly predicted internet addiction at T3 (*β* = 0.31, *p* =.002). Cross-lagged effects were also observed. Insomnia at T1 significantly predicted internet addiction at T2 (*β* = 0.23, *p* =.006), and insomnia at T2 significantly predicted internet addiction at T3 (*β* = 0.26, *p* =.005). Conversely, internet addiction at T1 significantly predicted insomnia at T2 (*β* = 0.21, *p* =.002), and internet addiction at T2 significantly predicted insomnia at T3 (*β* = 0.23, *p* =.002). These results suggest that within-person deviations in insomnia at each time point positively predicted within-person deviations in internet addiction at subsequent time points, and vice versa.

## Discussion

### Persistence and stability of insomnia and internet addiction

The RI-CLPM autoregressive paths revealed that T1 insomnia and internet addiction in college students significantly and positively predicted T2 insomnia and internet addiction. Similarly, T2 insomnia and internet addiction also predicted T3 insomnia and internet addiction with the same significance and positivity. These findings align with previous research [18, 23, 45]. The autoregressive patterns of insomnia and internet addiction suggest that these conditions do not diminish or disappear over time on their own. Instead, they may intensify due to various factors, such as academic pressure, social needs, and entertainment preferences [18, 45]. This underscores the persistence and stability of insomnia and internet addiction among college students [52]. Without external intervention or effective self-regulation mechanisms, insomnia and internet addiction are unlikely to alleviate on their own. Therefore, it is crucial to address these issues among college students early on, as timely intervention is essential to preventing the problem from worsening.

Regarding insomnia, it can be reasonably assumed that college students who experience insomnia at one time point are likely to continue experiencing it in subsequent periods, with the potential for further persistence. This autoregressive pattern can be understood through both neurobiological and behavioral pathways. Crucially, the daytime consequences of insomnia—such as fatigue, poor concentration, and emotional instability [51]—often initiate or exacerbate maladaptive coping behaviors (e.g., excessive napping, caffeine overuse, reduced daytime activity, heightened anxiety about sleep [33] and cognitive processes (e.g., rumination, catastrophic thinking about sleep loss) [46]. These behaviors and cognitions directly interfere with subsequent sleep onset and maintenance, thereby fueling the persistence of insomnia. While neurobiological studies also suggest that chronic insomnia is associated with alterations in brain circuits related to emotion and cognition [10], the behavioral reinforcement pathways provide a more direct explanation for the self-perpetuating cycle observed in our self-reported data.

Concerning internet addiction, when college students seek emotional catharsis and satisfaction from the internet after experiencing interpersonal problems or stressful events, it tends to reinforce their motivation to continue using the internet [26]. This habitual use, if prolonged, may lead to cognitive impairments in the brain’s reward and executive control systems [34]. These neural alterations are directly reflected in behavior as significantly lower self-control and greater sensitivity to rewards [50], which perpetuate maladaptive internet use by making it more difficult for students to manage cravings and impulsive online behaviors.

### Between-person relationship between insomnia and internet addiction

The analysis at the between-person level revealed no significant correlation between insomnia and internet addiction in this study. This finding suggests the absence of a stable trait-level association between the two constructs, aligning with etiological models proposing distinct dispositional pathways [9, 44]. For instance, trait-like insomnia is theorized to stem from neurobiological hyperarousal [44], whereas trait-like vulnerability to internet addiction is linked to reward-processing deficits [9]. This result is consistent with Zhang et al. [55] but inconsistent with other cross-sectional studies [35, 47]. Crucially, cross-sectional designs cannot disentangle trait-level associations from transient state co-occurrence, whereas our RI-CLPM explicitly isolates these levels [38]. Several factors, including sample selection, measurement tools, and data analysis methods, may account for these discrepancies. A key theoretical explanation, however, lies in their divergent underlying etiologies at the trait level.

The psycho-physiological-behavioral model of insomnia posits that trait-like insomnia arises from excessive stress reactivity, linking it to hyperarousal in individuals with specific genotypes or personality traits (e.g., neuroticism) [18]. Neurobiological evidence indicates that this trait manifests as hypothalamic-pituitary-adrenal axis dysregulation [11]. In contrast, Young’s [54] model conceptualizes trait-like vulnerability to internet addiction as a reward-processing deficit, akin to substance addiction, supported by fMRI studies showing altered resting-state network connectivity [20]. College students unable to fulfill intrinsic needs in real life may develop this vulnerability. Individuals may use the internet to alleviate negative emotions, which over time consolidates into addiction [27]. Therefore, while trait-like insomnia may reflect passive psychobiological dysregulation [11], trait-like internet addiction vulnerability may involve active compensatory behaviors [50]. This fundamental divergence in etiology explains the null between-person correlation: these distinct trait-level vulnerabilities may operate via independent pathways.

Importantly, this finding does not contradict our within-person findings: The significant cross-lagged effects indicate that state-level insomnia may trigger state-level internet use as a coping mechanism, forming a dynamic loop despite the underlying independence at the trait level [52].

### Reciprocal relationship between insomnia and internet addiction

The cross-lagged paths revealed a significant bidirectional relationship between insomnia and internet addiction, with each condition predicting the other over time. This finding aligns with Yao et al.’s [52] study using the cross-lagged panel model (CLPM), but our use of RI-CLPM provides stronger evidence that these effects primarily occur at the within-person level.

From a psychological perspective, insomnia may increase irritability, anxiety, and negative mood, which disrupt normal circadian rhythms and heighten nocturnal arousal [56]. Students who remain awake at night may turn to online activities—gaming, social media, or streaming videos—as a way to cope with emotional distress. While this behavior may offer temporary relief, it can develop into a maladaptive coping strategy that maintains or worsens insomnia [2]. Similarly, excessive internet use may impair emotion regulation and increase bedtime procrastination, further delaying sleep onset [57].

From a behavioral perspective, sleep deprivation caused by insomnia leads to fatigue and reduced self-control, which in turn makes it more difficult to limit time spent online. Prolonged screen exposure and engagement in stimulating online content can delay bedtime, disrupt circadian rhythms, and intensify physical exhaustion, thereby worsening insomnia symptoms [19]. This reciprocal pattern creates a vicious cycle in which insomnia and internet addiction perpetuate each other over time.

From a neurobiological perspective, the bidirectional relationship is supported by overlapping neural correlates. Recent neuroimaging evidence points to dysregulation within the default mode network and other circuits governing cognitive control and emotional regulation as a shared substrate that may underlie both conditions [6, 34]. These functional alterations provide a plausible basis for the impaired self-regulation and increased reward sensitivity that perpetuate the cycle.

### Practical implications

The findings of this study underscore the importance of concurrently addressing insomnia and internet addiction in real-world settings and provide actionable guidance for university mental health services and student wellness management. First, given the mutually reinforcing cycle between insomnia and internet addiction at the within-person level, simultaneous attention to sleep quality and internet use patterns should be incorporated into routine mental health screenings for university students. Identification of one problem should trigger a systematic assessment for the other. Second, university counseling centers and wellness departments are encouraged to integrate sleep hygiene education and healthy internet use strategies into health promotion initiatives and freshman orientation programs. These efforts may help students establish healthy behavioral patterns early, reducing the risk of problem escalation. Third, clinicians working with students should assess both sleep and internet use behaviors when treating either insomnia or internet addiction, ensuring that intervention plans explicitly address their co-occurrence and dynamic interplay in order to disrupt the vicious cycle.

### Limitations

Several limitations warrant consideration. First, while the RI-CLPM framework effectively disentangled within-person dynamics from between-person differences, our focused examination of the bidirectional insomnia-internet addiction relationship precluded investigation of underlying mechanisms. Data on potential mediating/moderating variables (e.g., maladaptive cognitions, emotion dysregulation, specific online activities, or academic stress) were not collected. Consequently, although we robustly demonstrate core temporal interplay, our findings cannot elucidate the psychological, behavioral, or neurobiological pathways driving this reinforcement.

Second, the intentional focus on this parsimonious model limited our capacity to examine broader contextual factors. Although we controlled for age and gender, the absence of socioeconomic status (SES) and other covariates (e.g., psychiatric comorbidities, substance use) represents a notable constraint. The failure to account for SES may introduce confounding bias at the between-person level, potentially compromising causal interpretation of observed associations. Future research should prioritize the inclusion of SES measures to allow for more comprehensive modeling.

Third, our sample was predominantly female (86.3%), which may restrict the generalizability of the findings. Although we statistically controlled for gender in all analyses, future studies should recruit more gender-balanced samples or examine potential gender-specific pathways. Finally, our exclusive reliance on self-reported measures may have introduced response biases, such as recall errors and social desirability effects. Future research would benefit from incorporating objective tools—such as accelerometers for sleep assessment [42] or digital trace data to capture internet use behaviors—to enhance measurement precision and strengthen the validity of the findings.

## Conclusion

The current study offers a deeper understanding of the relationship between insomnia and internet addiction among Chinese college students by utilizing a longitudinal design. A key finding was the bidirectional nature of this relationship: insomnia and internet addiction were found to positively predict each other over time. Furthermore, the study highlighted the advantages of using the random intercept cross-lagged panel model (RI-CLPM), an innovative methodology that allowed us to demonstrate that this association primarily operates at the within-person level, rather than between-persons. While the findings underscore the dynamic relationship between insomnia and internet addiction, further research is needed to explore potential mediators and moderators of this relationship among college students. These factors could provide valuable insights into the mechanisms underlying this comorbidity and inform targeted interventions.

## Data Availability

The data and analytical scripts are available via: https://osf.io/u3jxk/overview? view_only=6659c90cd8bc42468c1acf4be10425ba.
